# Lysine acetylation modulates mouse sperm capacitation

**DOI:** 10.1038/s41598-018-31557-5

**Published:** 2018-09-06

**Authors:** Carla Ritagliati, Guillermina M. Luque, Cintia Stival, Carolina Baro Graf, Mariano G. Buffone, Dario Krapf

**Affiliations:** 1 Laboratory of Cell Signal Transduction Networks, Instituto de Biología Molecular y Celular de Rosario (IBR), CONICET-UNR, Rosario, 2000 Argentina; 2 Laboratory of Cellular and Molecular Reproductive Biology, Instituto de Biología y Medicina Experimental (IBYME), CONICET, Buenos Aires, C1428ADN Argentina

## Abstract

Mammalian sperm are unable to fertilize the egg immediately after ejaculation. To gain fertilization competence, they need to undergo a series of modifications inside the female reproductive tract, known as capacitation. Capacitation involves several molecular events such as phosphorylation cascades, hyperpolarization of the plasma membrane and intracellular Ca^2+^ changes, which prepare the sperm to develop two essential features for fertilization competence: hyperactivation and acrosome reaction. Since sperm cells lack new protein biosynthesis, post-translational modification of existing proteins plays a crucial role to obtain full functionality. Here, we show the presence of acetylated proteins in murine sperm, which increase during capacitation. Pharmacological hyperacetylation of lysine residues in non-capacitated sperm induces activation of PKA, hyperpolarization of the sperm plasma membrane, CatSper opening and Ca^2+^ influx, all capacitation-associated molecular events. Furthermore, hyperacetylation of non-capacitated sperm promotes hyperactivation and prepares the sperm to undergo acrosome reaction. Together, these results indicate that acetylation could be involved in the acquisition of fertilization competence of mammalian sperm.

## Introduction

After ejaculation, mammalian sperm need to undergo a series of biochemical and physiological modifications inside the female reproductive tract in order to gain fertilization competence^1,2^. These changes, collectively known as capacitation, prepare the sperm to develop two main features that are essential for fertilization: hyperactivated flagellar motility and the ability to undergo a secretory event known as acrosomal exocytosis. In mammals, the capacitation process can be mimicked *in vitro* by sperm incubation in standard culture medium containing Ca^2+^, HCO_3_^−^, energy sources, and a cholesterol acceptor that is usually BSA. Upon sperm exposure to these conditions, one of the first signaling events observed is a fast increase of intracellular cAMP concentration, as a result of the stimulation of the soluble adenylyl cyclase (sAC), with the consequent PKA activation^3,4^. Other molecular events related to capacitation include: cholesterol loss from the sperm plasma membrane, increased membrane fluidity^5^, changes in ion concentrations^6^, increase of the intracellular pH^7^, hyperpolarization of the plasma membrane^8–10^ and the increase of protein tyrosine phosphorylation^11^.

During the entire lifetime of a mature sperm, its functions are maintained and regulated without protein biosynthesis. Sperm cells are transcriptionally and translationally silent and thus, mainly rely on post-translational modifications (PTM) to regulate physiological processes. Reversible phosphorylation of proteins has a crucial role for proper cellular functioning in all eukaryotic organisms, providing an efficient and rapid system to initiate or cease a biological response. The importance of phosphorylation in post-ejaculated sperm was recognized when tyrosine phosphorylation, dependent on PKA activation, was correlated to sperm capacitation more than 20 years ago^11^. On the other hand, acetylation of proteins, in spite of being as abundant and ubiquous as phosphorylation^12^, has not been much explored in sperm. Lysine acetylation is a reversible and highly regulated PTM that is known to play a key role in modulating several cellular processes. It involves the transfer of the acetyl group from acetyl-CoA to the ε-amino group of lysine residues, catalyzed by acetyltransferases (ATs), while the removal is carried out by deacetylases (DACs). According to functional criteria and their homology with yeast proteins, DACs are classified into four classes (I-IV), which can be further divided into Zn^2+^-dependent or classical (classes I, II and IV) (DACs 1–11) and NAD^+^-dependent or most commonly named sirtuins (class III, SIRT1-7)^13,14^. To study this PTM two deacetylase inhibitors have been widely used: Trichostatin A (TSA) inhibits all DACs roughly to the same extent^15^, while Nicotinamide (NAM) inhibits sirtuins because of their need for NAD^+^ as a co-substrate^16^.

Recently, two groups identified 456 and 576 acetylated proteins in non-capacitated^17^ and capacitated^18^ human sperm respectively. Between the non-capacitated and capacitated sperm, different acetylation profiles were observed in proteins involved in sperm capacitation, sperm-egg recognition, sperm-egg plasma fusion, and fertilization, indicating that acetylation may be required for sperm capacitation and fertilization^17^.

In the present work, we show an acetylation increase in mouse sperm proteins during the capacitation process. Pharmacological lysine acetylation of proteins in non-capacitated sperm promoted an increase in the phosphorylation status of PKA substrates, probably through acetylation of its subunits that prompted cAMP independent activation. In addition, hyperpolarization of the plasma membrane and increased intracellular Ca^2+^ concentration ([Ca^2+^]_i_) through CatSper opening were also observed. These molecular events are all associated with the capacitated sperm state, and took place even in the absence of HCO_3_^−^ and BSA. Furthermore, in correlation with these results, sperm aquired both hyperactivated motility and acrosomal responsiveness, pointing towards the importance of lysine acetylation in sperm physiology.

## Results

### Expression and localization of lysine-acetylated proteins in mouse sperm

**Figure 1:**
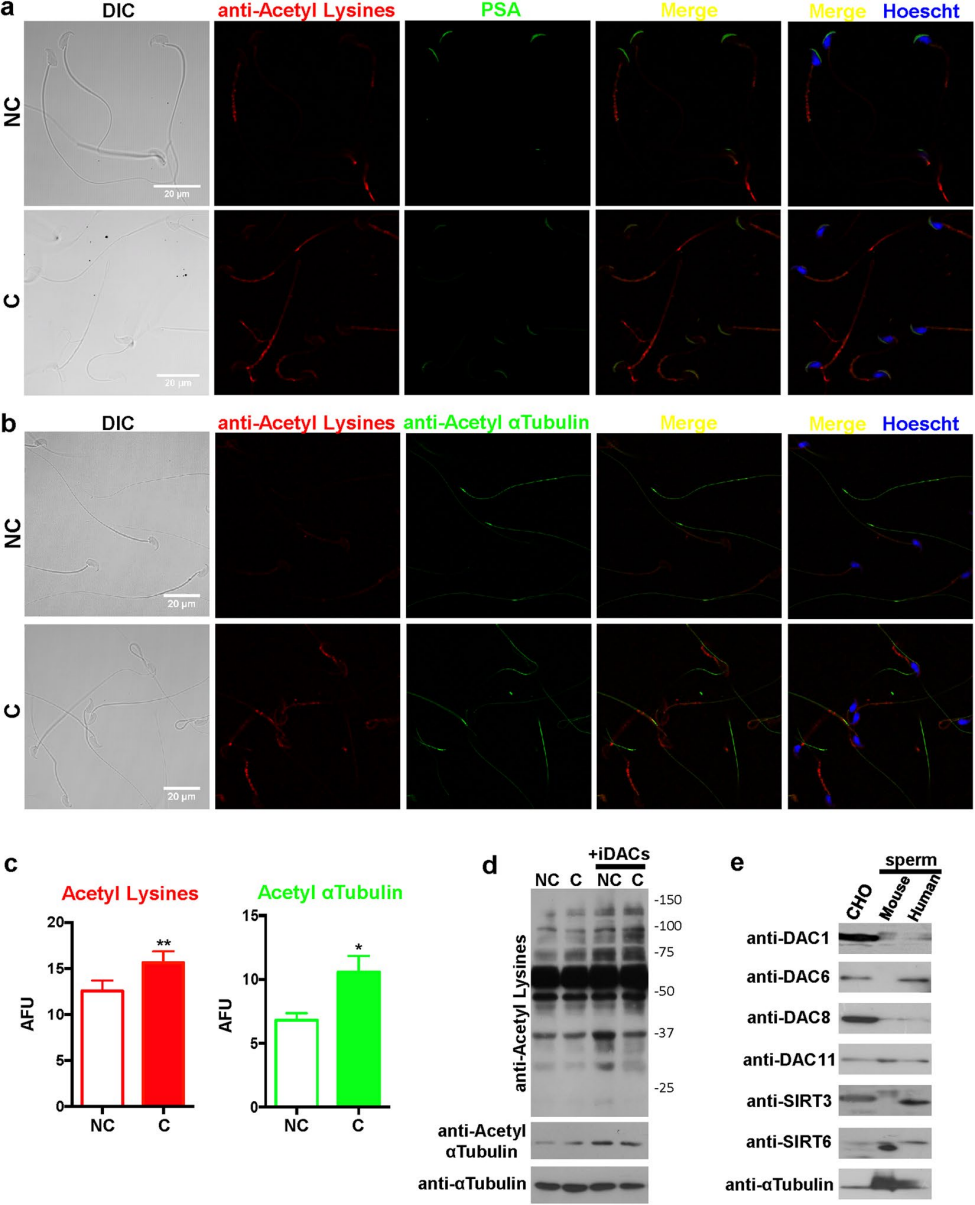
Acetylated proteins in mouse sperm. (**a**) Immunofluorescence of acetylated proteins (red) in non-capacitated (NC) and capacitated (C) sperm. Alexa 546 anti-rabbit was used as secondary antibody. PSA-FITC (green) was used as an acrosome marker and the DNA was stained with Hoescht (blue). Scale bar, 20 μm. DIC, differential interference contrast. The images were acquired with a Zeiss LSM880 confocal microscope using the same microscope settings for both conditions and processed with ImageJ. (**b**) Immunofluorescence of acetylated proteins (red) and acetylated αTubulin (green) in non-capacitated (NC) and capacitated (C) sperm. Alexa 546 anti-rabbit and Alexa 488 anti-mouse were used as secondary antibodies. The DNA was stained with Hoescht (blue). Scale bar, 20 μm. DIC, differential interference contrast. Images were acquired with a Zeiss LSM880 confocal microscope using the same microscope settings for both conditions and processed with ImageJ. (**c**) Fluorescence intensities (AFU: arbitrary fluorescence units) of acetylated proteins (red) and acetylated αTubulin (green) were quantified using ImageJ, in non-capacitated (NC) and capacitated (C) sperm. Data represent mean ± SEM from at least 100 sperm. ^*^P < 0.05, ^**^P < 0.005. (**d**) Western blot analysis of non-capacitated (NC) and capacitated (C) sperm in the absence or presence of deacetylase inhibitors (+iDACs) (5 μM TSA and 0.5 mM NAM) using anti-Acetyl Lysines, anti-Acetyl αTubulin and anti-αTubulin antibodies. (**e**) Western blot analysis of total protein extracts from *in vitro* CHO cell cultures, mouse sperm and human sperm, with antibodies against the following deacetylases: DAC1, DAC6, DAC8, DAC11, SIRT3 and SIRT6. anti-αTubulin was used as a loading control. Molecular weights are indicated in kDa. Full-length blots are presented in Fig. S1.

Despite increasing evidence involving lysine acetylation as an important PTM in different cell types, the presence of lysine acetylated proteins in mature mouse sperm and its role during capacitation has not been explored yet. Immunofluorescence analysis using antibodies that detect acetylated proteins on the ɛ-amino group of lysine residues (anti-Acetyl Lysines) was performed on both non-capacitated and capacitated sperm (Fig. 1a) and showed wide distribution of acetylated proteins mostly in the mid-piece, but also in the acrosome and principal piece. Acetylation of Lys40 in αTubulin is known to be a predominant and highly conserved PTM in ciliated cells. Mouse sperm are no exception since acetylation of αTubulin is necessary for their appropriate morphology and motility, and required for normal sperm flagellar function^19^. In order to rule out that the lysine acetylation detected corresponded solely to αTubulin, sperm were co-stained with anti-Acetyl Lysines and anti-Acetyl αTubulin antibodies. As Fig. 1b shows, acetylated αTubulin was only present in the tail, showing a variable degree of acetylation among different cells. Interestingly, the quantification of fluorescent signals shows a significant increase in the acetylation levels of capacitated sperm (Fig. 1c). This result is in agreement with acetylproteome studies of human sperm^17,18^, where an increase in acetylated sites of proteins important for sperm-specific functions is observed when incubated under capacitating conditions. Western blot of protein extracts from non-capacitated and capacitated sperm with anti-Acetyl Lysines antibody revealed a smear-like staining across all molecular weights, which showed lysine acetylation of a large number of proteins spanning a wide mass range (Fig. 1d). Incubation of sperm with two deacetylase inhibitors (iDACs), Trichostatin A (TSA) and Nicotinamide (NAM), increased the acetylation detected with both anti-Acetyl Lysines and anti-Acetyl αTubulin antibodies (Fig. 1d). Thus, we were able to induce pharmacological hyperacetylation of sperm and validated the specificity of the antibodies used in these experiments. In addition, several deacetylases were detected by western blot in mouse sperm: DAC1, DAC8, DAC11, SIRT3 and SIRT6 (Fig. 1e), validating the use of deacetylase inhibitors. Since some deacetylases have already been identified in human sperm^17,20^, they were used as a control as well as Chinese hamster ovary (CHO) cells.

### Hyperacetylation promotes phosphorylation of PKA substrates

**Figure 2:**
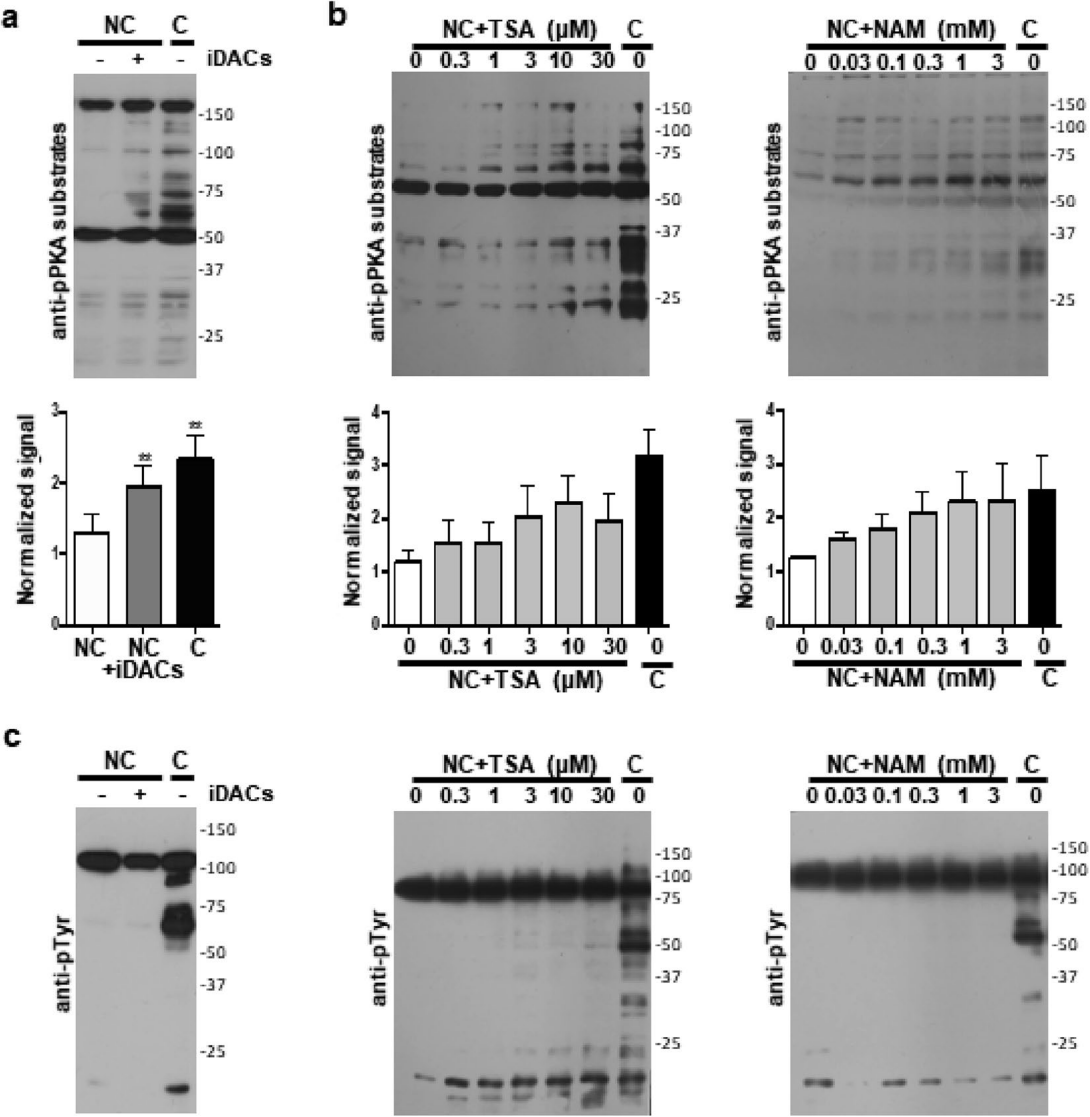
Effect of deacetylases inhibition on capacitation-associated phosphorylation cascades. (**a**,**b**) Western blot analysis using anti-pPKA substrates antibody and quantification of anti-pPKA substrates signal for each condition using tyrosine phosphorylated Hexokinase to normalize. Data represent mean ± SEM from three independent experiments. Paired Student’s *t* test was performed to compare each condition with the NC: ^**^P < 0.005. (**a**) Sperm were incubated in non-capacitating media (NC) in the absence (−) or presence (+iDACs) of deacetylase inhibitors (5 μM TSA and 0.5 mM NAM), and in capacitating media (C). (**b**) Sperm were incubated in non-capacitating media (NC) with increasing concentrations of the deacetylase inhibitors Trichostatin A (TSA) and Nicotinamide (NAM), and in capacitating media (C). (**c**) Western blots with anti-pTyr antibody of the conditions tested in a and b. Molecular weights are indicated in kDa. Tyrosine phosphorylated Hexokinase (95 kDa) served as a loading control^11^.

One of the first events associated to the onset of capacitation is activation of PKA, which can be analyzed using a monoclonal anti-phospho-PKA substrate antibody (anti-pPKA substrate). This antibody detects phosphorylation on serine or threonine residues at positions Arg-Arg-X-pSer/pThr (corresponding to a PKA consensus phosphorylation sequence) and has been previously used in sperm from different species, including mouse^21–23^. Moreover, increasing evidence suggests a role of lysine acetylation as a regulator of PKA activity^24^. Thus, in order to study the effect of pharmacological hyperacetylation on the phosphorylation of PKA substrates, we incubated sperm cells in non-capacitating conditions, in the absence or presence of iDACs. Western blots with anti-pPKA substrates antibody showed an increase in the phosphorylation of PKA substrates in non-capacitating conditions supplemented with two deacetylase inhibitors, TSA and NAM (iDACs) (Fig. 2a), as well as in the control capacitating condition. As seen in Fig. 2b, this increase was dose-dependent for both TSA and NAM. It is worth noticing that even though PKA activity, which is known to be upstream of tyrosine phosphorylation, was detected; no tyrosine phosphorylation was promoted by iDACs in non-capacitating medium (Fig. 2c).

**Figure 3:**
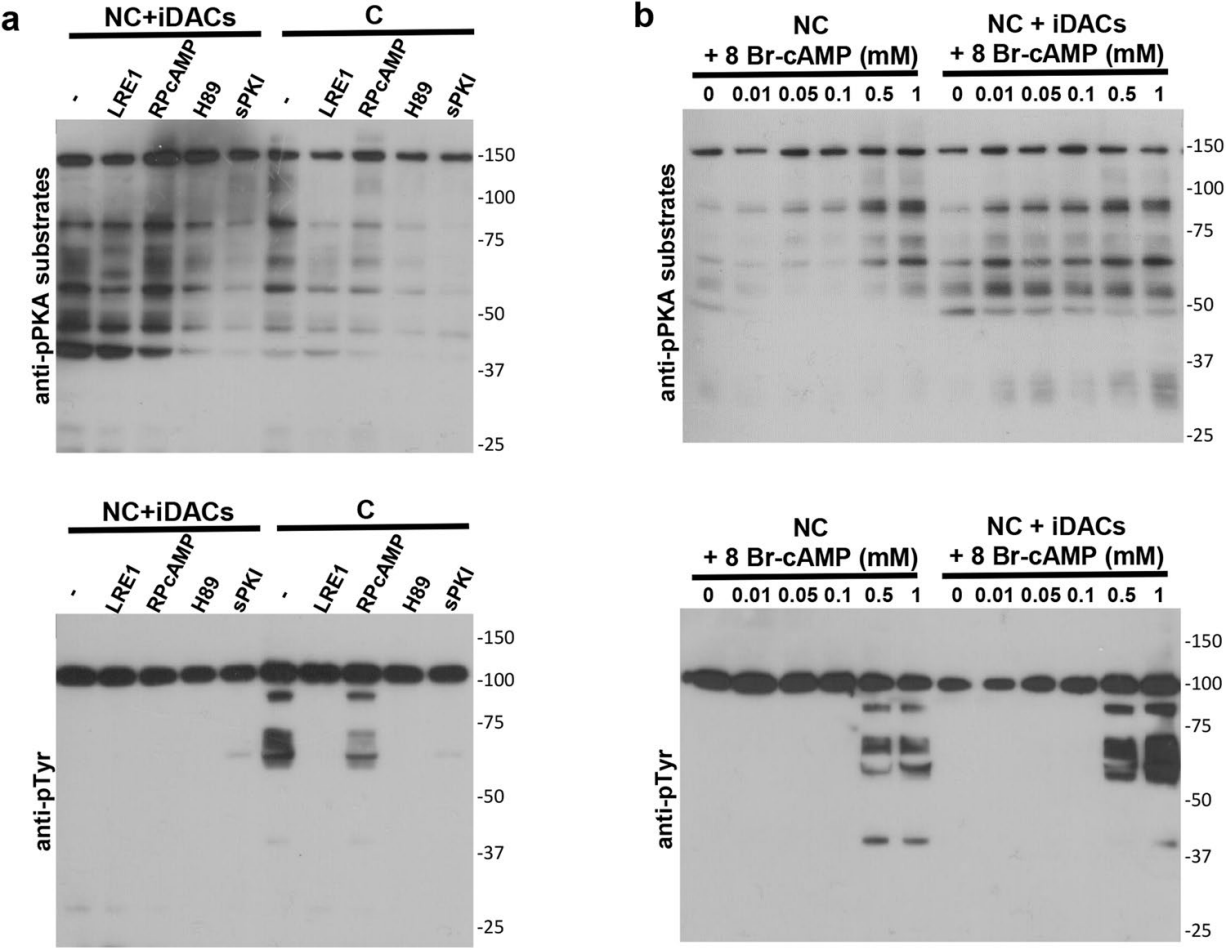
PKA is regulated by lysine acetylation. Western blot analysis using anti-pPKA substrates and anti-pTyr antibodies. (**a**) Sperm were pre-incubated in non-capacitating media with the following inhibitors: LRE1 (50 μM), Rp-cAMPS (0.5 mM), H89 (30 μM) and sPKI (30 μM), before incubation in non-capacitating media (NC) with deacetylase inhibitors (NC + iDACs) (5 μM TSA and 0.5 mM NAM) or capacitating media (C), for 60 min. (**b**) Sperm were incubated in non-capacitating media (NC) supplemented with BSA (5 mg/ml), IBMX (0.1 mM) and increasing concentrations of 8Br-cAMP, in the absence (−) or presence (+iDACs) of deacetylase inhibitors. Molecular weights are indicated in kDa. Tyrosine phosphorylated Hexokinase (95 kDa) served as a loading control^11^.

The Ser/Thr kinase PKA is a tetramer composed of two regulatory subunits (PKARII in mouse sperm) that bind and inactivate two catalytic subunits (PKAc α2 in mouse sperm)^25^. Upon binding of cAMP, the regulatory subunits release the active catalytic subunits. An increase in PKA substrates phosphorylation can be promoted by a direct or indirect (through cAMP production) activation of PKA, or by inhibition of Ser/Thr phosphatases^26^. In order to address these possibilities, sperm were pre-incubated with LRE1 (an inhibitor of sAC)^27^, Rp-cAMPS (binds to PKARII cAMP binding site, preventing the release of active PKAc), H89 (inhibits PKAc by competing with ATP) or sPKI (a pseudo-substrate that inhibits PKAc by blocking its active site), before addition of non-capacitating media containing iDACs or capacitating media. Western blot analysis showed that while all these inhibitors diminished the phosphorylation levels of PKA substrates when present in capacitating media, only those inhibitors that directly act on PKAc, H89 and sPKI, affected phosphorylation of PKA substrates in the presence of iDACs (Fig. 3a). These results indicate that neither inhibition of cAMP synthesis, nor blockade of cAMP binding to PKARII have any effect on the PKA activity promoted by iDACs, suggesting that PKAc activates independently of cAMP when hyperacetylation is induced. To test this hypothesis, sperm were incubated in non-capacitating media with the phosphodiesterase inhibitor IBMX and increasing concentrations of the permeable cAMP agonist 8Br-cAMP, in the absence or presence of iDACs. As already described^28^, in the absence of iDACs, phosphorylation of PKA substrates depends on the concentration of 8Br-cAMP, with a maximum observed with 0.5–1 mM, which correlates to the promotion of Tyr phosphorylation. On the other hand, in the presence of iDACs, PKA activation resulted independent of 8Br-cAMP concentration, since the lowest concentration assayed (10 µM) gave maximum activity (Fig. 3b). Even though phosphorylation of PKA substrates was observed in all conditions assayed, Tyr phosphorylation still required over 0.5 mM of 8Br-cAMP (Fig. 3b), indicating that phosphorylation of PKA substrates is not sufficient to promote Tyr phosphorylation.

**Figure 4:**
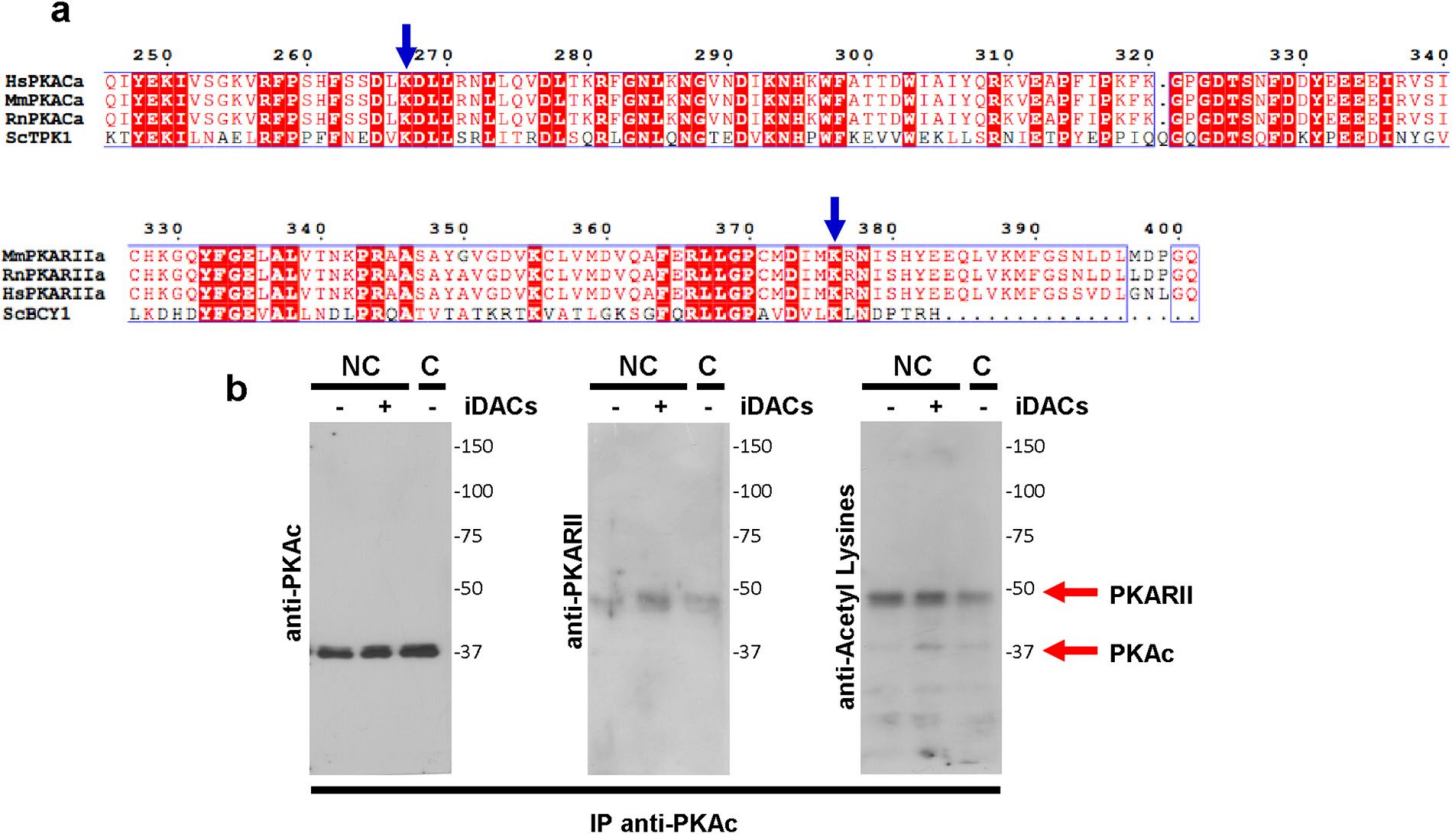
PKA acetylation. (**a**) PKA sequence alignment of catalytic α (PKACa) and type 2 regulatory α (PKARIIa) subunits from human (Hs), mouse (Mm), rat (Rn) and *Saccharomyces cerevisiae* (Sc) were aligned using Clustal X2.1 and edited online with ESPript 3.0. Conserved amino acids are in white highlighted in red. The acetylated lysines previously identified in human sperm^18^ are indicated with blue arrows. (**b**) Protein extracts from sperm incubated in capacitating (**c**) or non-capacitating (NC) conditions in the absence (−) or presence (+) of iDACs (5 μM TSA and 0.5 mM NAM) were subjected to immunoprecipitation with anti-PKAc antibodies. Immunoprecipitates were analyzed by western blots with anti-PKAc (left panel), anti-PKARII (middle panel) and anti-Acetyl Lysines (right panel) antibodies. Molecular weights are indicated in kDa.

During canonical activation of PKA, binding of cAMP to PKARII triggers a conformational change that lowers its affinity towards PKAc, releasing enzymatic PKA activity. The same effect is plausible for any PTM on PKA subunits that triggers a conformational change resulting in decreased affinity between subunits. Recently, it has been shown in mammalian cell cultures that hyperacetylation, through inhibition of deacetylases with TSA and NAM, as well as through overexpression of acetyltransferases, reduces PKAR-PKAc affinity, releasing active PKAc^24^. Furthermore, as reported in the acetylproteome studies of human sperm, both PKAc and PKARII appear to be acetylated in Lys267 and Lys379 respectively, only in capacitated sperm^17,18^. As seen in Fig. 4a, the sequence alignment of catalytic (PKACa) and regulatory (PKARIIa) PKA subunits from human, mouse, rat and yeast shows that lysines found to be acetylated in capacitated human sperm are conserved in both PKA subunits of mouse sperm. In order to confirm PKA acetylation, immunoprecipitation with anti-PKAc antibody was performed. Western blot analyses show that PKARII co-immunoprecipitates with PKAc and that both subunits are acetylated in the conditions tested (Fig. 4b).

### Hyperacetylation induces membrane hyperpolarization

**Figure 5:**
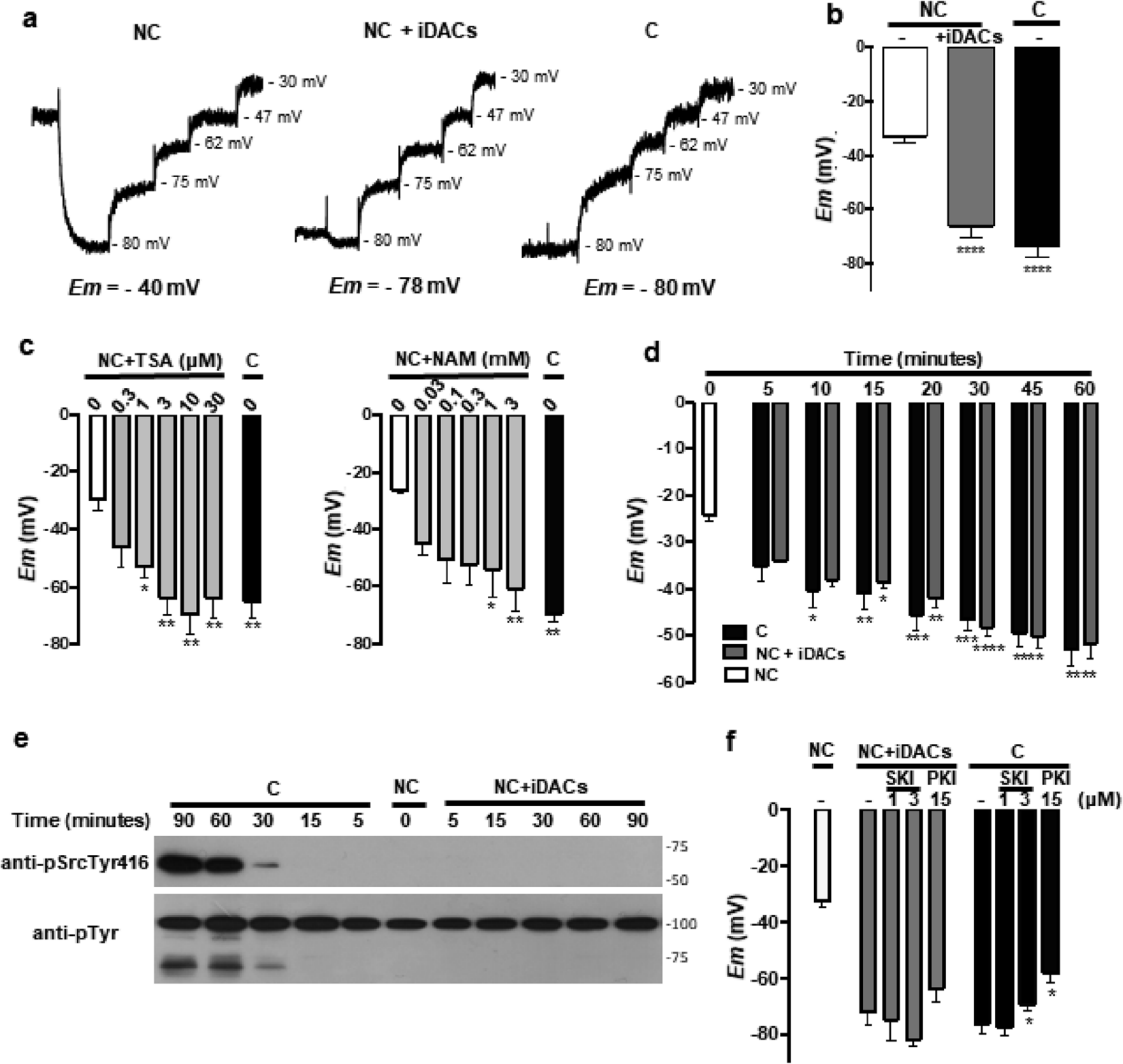
Hyperacetylation-induced hyperpolarization. Data represent mean ± SEM from at least three independent experiments. (**a**,**b**) Sperm were incubated under capacitating (C) or non-capacitating (NC) conditions in the absence (−) or presence (+iDACs) of iDACs (5 μM TSA and 0.5 mM NAM). (**a**) Representative fluorescence traces showing the values of sperm *Em*. (**b**) *Em* measurements. Paired Student’s *t* test between each condition and the NC (white bar): ^****^P < 0.0001. (**c**) *Em* measurements of sperm incubated under capacitating (C) or non-capacitating (NC) conditions with increasing concentrations of either TSA or NAM. One-way ANOVA with Dunnett’s multiple comparisons test was performed. Statistical significance with respect to the non-capacitating control condition (white bar): ^*^P < 0.05, ^**^P < 0.005. (**d**) *Em* measurements of sperm incubated in capacitating (C, black bar) or non-capacitating conditions with iDACs (NC + iDACs, grey bar) (5 μM TSA and 0.5 mM NAM) for different times. One-way ANOVA with Turkey’s multiple comparisons test was performed. Statistical significance with respect to the non-capacitating control condition (time 0, white bar): ^*^P < 0.05, ^**^P < 0.005, ^***^P < 0.0005, ^****^P < 0.0001. There was no significant difference between conditions within each time point. (**e**) Western blot analysis using anti-pSrcTyr416 and anti-pTyr of sperm incubated in capacitating (C) or non-capacitating conditions with iDACs (NC + iDACs) (5 μM TSA and 0.5 mM NAM) for different times. Molecular weights are indicated in kDa. Tyrosine phosphorylated Hexokinase (95 kDa) served as a loading control^11^. Full-length blots are presented in Fig. S2. (**f**) *Em* measurements of sperm incubated for 60 min in capacitating (C, black bars) or non-capacitating conditions in the absence (NC, white bar) or presence of iDACs (NC + iDACs, grey bars) (5 μM TSA and 0.5 mM NAM), after 10 min pre-incubation with the Src inhibitor SKI606 (1–3 μM SKI) or 15 μM sPKI. Paired Student’s *t* test was performed to compare each condition without (-) or with the inhibitors (SKI/PKI): ^*^P < 0.05.

Capacitation-induced hyperpolarization is key to enabling mouse sperm to undergo the acrosome reaction^8^. To test whether acetylation regulates membrane potential (*Em*), we performed a population assay using the carbocyanine dye DISC_3_(5). Treatment with iDACs induced *Em* hyperpolarization (Fig. 5a,b), in a concentration-dependent manner on both TSA and NAM (Fig. 5c), and with the same kinetics as in capacitating media (Fig. 5d).

We have shown previously that Src kinase is the connecting player between PKA activation and hyperpolarization through SLO3 potassium channel regulation^9^. In order to determine if the regulation of *Em* by acetylation follows the same mechanism, we analyzed the phosphorylated state of Tyr416 of Src, which is a marker of its activation, both in non-capacitating media supplemented with iDACs and in capacitating media, at different time points. Figure 5e shows that although PKA is active upon hyperacetylation, phosphorylation of Tyr416-Src is not detected, suggesting that Src remains inactive. To further rule out the involvement of Src in the acetylation-induced hyperpolarization, we used Src inhibitor SKI606 (aka bosutinib), which is known to block the hyperpolarization triggered by capacitating media^9^. In agreement with the lack of Src activation, SKI606 did not block the *Em* hyperpolarization promoted by iDACs (Fig. 5f). Finally, in order to determine if this hyperpolarization is mediated by acetylation-activated PKA, we performed the assay in the presence of sPKI (Fig. 5f). Surprisingly, PKAc inhibition did not affect this process indicating that PKA is not essential for the hyperacetylation-induced hyperpolarization.

### Pharmacological hyperacetylation in non-capacitating media promotes calcium influx through CatSper opening, hyperactivation and acrosome reaction

**Figure 6:**
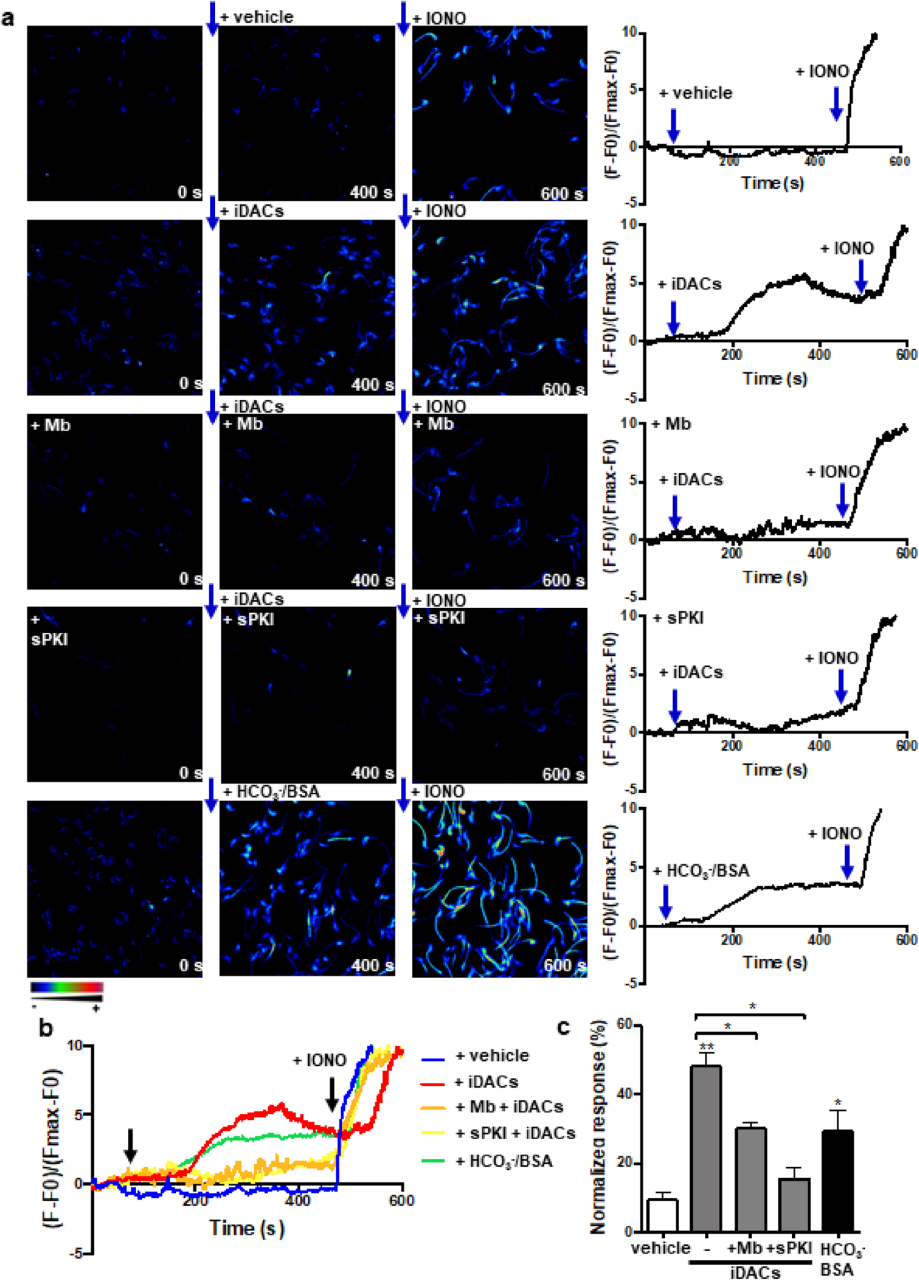
Hyperacetylation promotes Ca^2+^ influx. (**a**,**b**) Intracellular Ca^2+^ responses obtained before (0 s) and after (400 s) the addition of NC medium with vehicle (white bar), iDACs (grey bars) or HCO_3_^−^/BSA (black bar), and after (600 s) ionophore A23187 (IONO, 20 μM) treatment. Mibefradil (5 μM) or sPKI (30 μM) were added 10 min before starting the recording. (**a**) Representative fluorescence images of 5 independent experiments. Right traces show single cell [Ca^2+^]_i_ recordings obtained during each experiment. Arrows indicate the time of the additions. The color scale from red to blue is shown, where red represents the highest and blue the lowest intracellular Ca^2+^ concentrations. (**b**) Overlapped fluorescence traces of the 5 conditions tested. (**c**) Percentage of [Ca^2+^]_i_ increase induced under the indicated condition with respect to the increase induced by the ionophore (100%). Data represent mean ± SEM from at least three independent experiments. Statistical significance with respect to the non-capacitating control condition (white bar) using paired Student’s *t* test: ^*^P < 0.05, ^**^P < 0.005; and between the indicated + iDACs conditions, with and without mibefradil (Mb) or sPKI (grey bars): ^*^P < 0.05.

Calcium signaling is essential for all cell types. During capacitation there is an increase in [Ca^2+^]_i_, which is fundamental for hyperactivation and the acrosome reaction. It is well known that the sperm swimming behavior is controlled by rises in [Ca^2+^]_i_ that changes the flagellar beat pattern, and that the acrosome reaction is a Ca^2+^-mediated process^29^. Thus, we aimed to analyze Ca^2+^ fluxes under pharmacological hyperacetylation through intracellular single-cell Ca^2+^ imaging of Fluo3-AM–loaded spermatozoa incubated in conditions that do not support capacitation. Addition of non-capacitating media with iDACs increased [Ca^2+^]_i_ as did the capacitating media (Fig. 6a–c and Supplemental movies 1–5), while non-capacitating media did not. Interestingly, the hyperacetylation-driven Ca^2+^ increase was reduced with both mibefradil (T-type Ca^2+^ channel inhibitor) and sPKI. The bar graph in Fig. 6c summarizes the intracellular Ca^2+^ increases, in response to different treatments, normalized to the one obtained with the Ca^2+^ ionophore A23187 at the end of each experiment.

**Figure 7:**
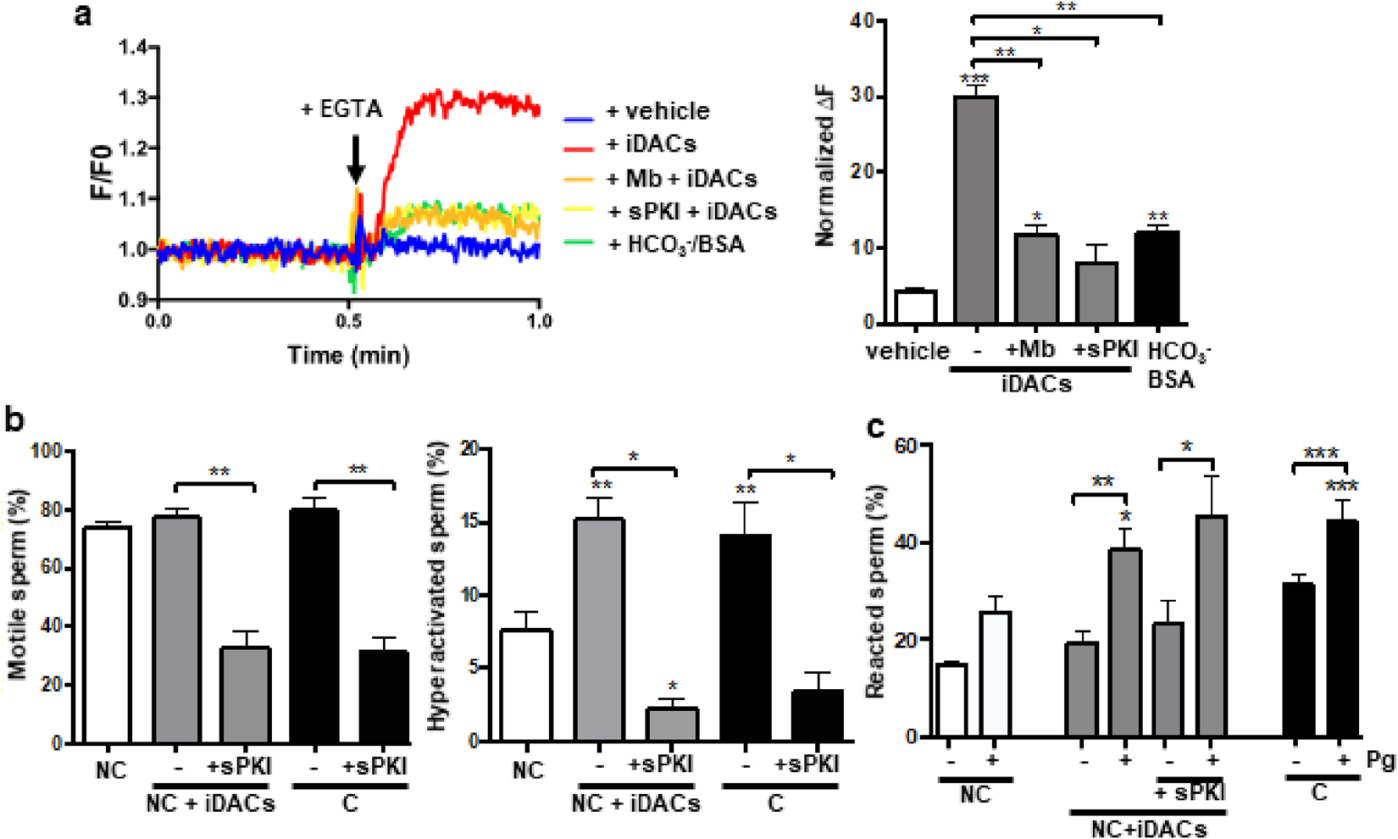
Functional relevance of acetylation in sperm physiology. (**a**) CatSper opening assessed by *Em* of sperm loaded with 1 μM DISC_3_(5). Representative recordings are shown for 5 conditions (left panel). Responses are expressed as (F_EGTA_ − F0)/F0 × 100 (right panel), where F_EGTA_ represents fluorescence intensity after 3.5 mM EGTA addition and F0 is the mean of 1 min of acquisition before addition of EGTA. Data represent mean ± SEM from at least three independent experiments. Statistical significance with respect to the non-capacitating control (white bar) and between the indicated conditions using paired Student’s *t* test: ^*^P < 0.05, ^**^P < 0.01, ^***^P < 0.0005. (**b**) Sperm were incubated for 60 min under capacitating (C, black bars) or non-capacitating conditions in the absence (NC, white bars) or presence of iDACs (NC + iDACs, grey bars). sPKI (30 μM) was added for 10 min before the addition of either C or NC media with iDACs. The percentage of motile and hyperactivated sperm were obtained using a CASA software. Data represent mean ± SEM from at least 4 independent experiments. Statistical significance of each condition with respect to the non-capacitating control (white bar) and between the two indicated conditions (−/+ sPKI) using paired Student’s *t* test: ^*^P < 0.05, ^**^P < 0.005. (**c**) After 60 min of incubation in the indicated conditions, cells were further incubated for 30 min in the absence (−) or presence (+) of 20 μM progesterone (Pg). sPKI (30 μM) was added for 10 min before the addition of Pg. The percentage of acrosome reacted sperm was assessed by FITC-PSA staining as described in Methods. Data represent mean ± SEM from at least 3 independent experiments. Statistical significance of each + Pg condition with respect to the non-capacitating control (white bar) and between the two indicated conditions (−/+ Pg) using paired Student’s *t* test: ^*^P < 0.05, ^**^P < 0.005, ^***^P < 0.001.

In extracellular medium deprived of Ca^2+^ and Mg^2+^, CatSper becomes permeable to Na^+^, which can be clearly evidenced by the Na^+^- dependent depolarization upon Ca^2+^ chelation^30,31^. The magnitude of this depolarization depends on the extent of CatSper opening. Therefore, by studying sperm *Em* in population assays using the fluorophore DISC_3_(5), we addressed whether hyperacetylation activates CatSper. In capacitated sperm, CatSper opening was evidenced by *Em* depolarization. When sperm were incubated in non-capacitating media with iDACs, depolarization was observed to a much higher extent, while no depolarization was detected in non-capacitated sperm. Consistent with the Ca^2+^ influx results, pre-incubation of sperm with mibefradil and sPKI diminished the pharmacological hyperacetylation-induced Catsper opening (Fig. 7a).

Aiming to investigate the biological importance of acetylation in sperm physiology, motility and acrosome reaction were evaluated. To this end, sperm cells were incubated in non-capacitating media in the absence or presence of iDACs and in capacitating control media. As seen in Fig. 7b, pharmacological hyperacetylation did not affect the total motility of the cells, but it did significantly increase the percentage of hyperactivated cells. As expected, considering the importance of [Ca^2+^]_i_ rises in this event, the inhibition of PKA with sPKI completely abrogated not only hyperactivation, but also total sperm motility (Fig. 7b). In order to evaluate acrosomal responsiveness, after 60 minutes of incubation in the three described experimental conditions, the cells were further incubated for 30 minutes in the absence or presence of progesterone (Pg). In agreement with hyperpolarization sufficiency for the acrosome reaction^8^, hyperacetylation promoted an increase in the induced acrosome reaction (Fig. 7c), which was not affected by PKAc inhibition. Altogether, these results point towards the importance of Lys acetylation in sperm physiology.

## Discussion

Our knowledge of the signaling cascade of capacitation is enriched by decades of studies involving mostly phosphorylation, ion fluxes and lipid modifications. This has led to a wide production of work, where PTM of sperm proteins, specifically phosphorylation in both tyrosine and serine/threonine residues have been studied the most^32^. On the other hand, acetylation of proteins, in spite of being an abundant and ubiquous PTM^12^, has not been much explored in sperm. Lysine acetylation is a conserved protein PTM first described in histones^33^ and for decades, it was thought to be exclusively a nuclear modification. Recent advances in the identification and quantification of lysine acetylation by mass spectrometry have increased our understanding of this PTM, implicating it in many biological processes through the regulation of protein interactions, activity and localization^34^.

Recently, two groups identified several acetylated proteins in human sperm^17,18^. While some proteins were acetylated, others were deacetylated during the capacitation process, pointing towards a highly dynamic PTM. Accordingly, using anti-Acetyl Lysines antibodies, we showed that several proteins are acetylated both in non-capacitated and capacitated sperm, being acetylation more abundant in the capacitated state. In media devoid of HCO_3_^−^, where there is no stimulation of sAC^35^ and hence, no cAMP production, PKA is inactive. Interestingly, pharmacological hyperacetylation, using two different iDACs, induced PKA activity even in the absence of HCO_3_^−^. Furthermore, when the sAC inhibitor LRE1 was used or when the cAMP binding sites on the regulatory PKA subunits were titrated with Rp-cAMPS, no inhibitory effect was observed in the phosphorylation of PKA substrates of sperm exposed to iDACs, indicating that activation of PKA was independent of a cAMP increase. A plausible explanation could reside in a sudden affinity drop between the regulatory and catalytic subunits. Canonical activation of PKAc involves a decreased affinity of PKARII for PKAc, promoted by cAMP binding to PKARII, releasing active PKAc. However, any other conformational change of either PKAR or PKAc, independent of cAMP, could also cause a reduction in their interaction. This is in agreement with the recent findings by Filteau *et al*.^24^, in which they show that inhibition of deacetylases with TSA or NAM, as well as overexpression of acetyltransferases, reduce the formation of the PKARII:PKAc complex, thus releasing active PKAc^24^. Furthermore, as reported in the acetylproteome studies from human sperm, both PKAc and PKARII are acetylated during capacitation^17,18^, and the acetylated residues (Lys267 and Lys379 for PKAc and PKARII respectively) are conserved in mouse sperm subunits. PKARII subunits consist of a dimerization and docking domain (D/D domain) located at the N terminus, followed by a flexible linker region that contains an inhibitory site, which resembles a peptide substrate and docks to the active site of PKAc, and two tandem universal cAMP-docking domains (CNBA and CNBB) at the C terminus^36^. Lys379 in PKARII is in the CNBB domain, therefore, its acetylation could result in a conformational change analogous to that induced by cAMP binding, or alternatively in an increased cAMP sensibility allowing PKA activation at lower cAMP levels. Regarding Lys267 in PKAc, although it does not locate to the active site, it has been shown by Yang *et al*.^37^ that mutagenesis of a near-by lysine (Lys285) reduced the interaction with PKARII subunits, indicating that a non-catalytic core lysine is involved in the inhibitory interaction. Thus, acetylation of these lysines could be regulating PKAR-PKAc association. Whether these PTM are indeed responsible for affinity loss between PKA subunits in sperm awaits exciting future work.

Activation of PKA results fundamental to trigger many aspects of the capacitation process. Among these, it has been shown that the PKA/Src/SLO3 axis regulates hyperpolarization of the mouse sperm plasma membrane^9^. Interestingly, *Em* hyperpolarization was observed upon pharmacological hyperacetylation in non-capacitating media. However, neither Src nor PKA were involved in this process, since their inhibition did not impair the acetylation-induced hyperpolarization. These results suggest that in addition to SLO channels regulation by phosphorylation^38^, lysine acetylation could also be modulating its activity. Consistent with this hypothesis, SLO3 α subunit sequence analysis using a lysine acetylated site predictor (PAIL, http://bdmpail.biocuckoo.org/.) shows an enrichment of acetylated lysines (more than 30) in its cytoplasmic C-terminal extension, called the gating ring, which allows channel gating to be altered in response to direct sensing of different intracellular ions, and by other second-messenger systems. In agreement with sperm *Em* hyperpolarization sufficiency for acrosomal responsiveness^8^, hyperacetylated sperm were capable of undergoing the acrosome reaction when challenged with progesterone. Moreover, acrosomal responsiveness was not impaired by PKA inhibition, further substantiating the sufficiency of *Em* hyperpolarization for the acrosomal exocytosis.

On the other hand, tyrosine phosphorylation of sperm proteins, which has been for many years considered the marker of capacitation, was not promoted by hyperacetylation. It is worth mentioning that while PKA activation occurs at the beginning of capacitation, tyrosine phosphorylation requires more than 30 minutes to be observed^23^, suggesting that although PKA activation is necessary for tyrosine phosphorylation to occur, it might not be sufficient and other molecular events that are not regulated by acetylation could be needed. Consistent with the results presented in this manuscript and in spite of being an accepted marker of sperm capacitation, tyrosine phosphorylation was recently shown not to be necessary for fertilizing capacity^39^.

In all cell types, Ca^2+^ plays essential roles as second messenger controlling several cellular processes. In sperm, apart from its fundamental role in phosphorylation signaling pathways, Ca^2+^ is also required for hyperactivation^29,40^. However, little is known about the mechanisms involved in controlling [Ca^2+^]_i_ as well as the identity of Ca^2+^ targets inside the sperm. The sperm-specific ion channel responsible for major sperm Ca^2+^ elevations and required for male fertility in mice was determined in 2001 with the cloning of CatSper1^41^. Since then, nine CatSper subunits composing the heteromeric CatSper channel have been identified and, at least five of them, have been shown to be indispensable for proper channel formation and function^42–49^. Mice lacking any of the α subunits (CatSper 1–4) or the accessory subunit δ, are all infertile due to an inability of spermatozoa to hyperactivate^50^. Humans with mutations within the CatSper1 or CatSper2 genes were also shown to be infertile^51,52^. CatSper current is weakly voltage-dependent and potently activated by intracellular alkalinization. Progesterone, a major steroid hormone released by the ovaries and the cumulus cells surrounding the egg, induces robust Ca^2+^ influx into both human and mouse sperm cells^53–55^. However, progesterone activates human CatSper, but not its murine counterpart^56^. Therefore, the exact mechanism of CatSper activation in mouse sperm is yet to be elucidated. Capacitating medium promotes CatSper opening and Ca^2+^ influx^57^. Interestingly, non-capacitating medium supplemented with iDACs induced higher responses, which were inhibited with mibefradil, indicative of the role of CatSper in this flux, as well as with sPKI. The fact that there was more Ca^2+^ influx and CatSper opening in the presence of hyperacetylation, a condition in which phospho-PKA substrates phosphorylation was not as high as with capacitating medium, implies that in addition to PKA phosphorylation, acetylation could also be directly or indirectly regulating CatSper. In this regard, the four α subunits (CatSper 1–4) from both human and mouse, posses lysines in their cytoplasmic domains predicted to be acetylated by two online site predictors (PLMLA, https://omictools.com/plmla-tool and PAIL, http://bdmpail.biocuckoo.org/). However, further evidence is needed in order to confirm this possibility.

Notably, sperm hyperacetylation in non-capacitating media also resulted in hyperactivation. Thus, both physiological capacitation-associated events, acrosomal responsiveness and hyperactivation, were achieved when sperm were pharmacologically hyperacetylated in the absence of HCO_3_^−^ and BSA. These data point towards a key role of this PTM in sperm capacitation, opening a new area of research in this field.

## Methods

### Chemicals and Reagents

Chemicals were obtained from the following sources. BSA (fatty acid-free), the adenylyl cyclase inhibitor LRE1 and the Ca^2+^ ionophore A23187 were purchased from Sigma. Protein kinase inhibitor PKI 14–22 amide myristoylated (sPKI) was obtained from Tocris. All other chemicals were purchased from Cayman Chemicals (Ann Arbor, MI). Anti-Acetyl αTubulin and the antibodies against the deacetylases were obtained from Santa Cruz Biotechnology. Anti-phosphotyrosine (anti-pTyr) monoclonal antibody (clone 4G10) was obtained from Upstate Biotechnology (Lake Placid, NY). Rabbit monoclonal anti-phospho-PKA substrates (anti-pPKA substrates) (clone 100G7E), anti-Acetyl Lysines (9441 S) antibodies and Horseradish peroxidase-conjugated anti-mouse and anti-rabbit IgG, were purchased from Cell Signaling Technology (Danvers, MA). Alexa 546 anti-rabbit, Alexa 488 anti-mouse and Slow-Fade Light reagents were obtained from Molecular Probes (Eugene, OR). Anti-PKAc was purchased from BD Biosciences (clone 5B) and anti-PKARII from Abcam (#38949). Anti-mouse and anti-rabbit IgG light chain (211-032-171 and 115-035-174 respectively) were purchased from Jackson ImmunoResearch Laboratories (West Grove, PA).

### Mouse Sperm Preparation

Experiments involving animals were conducted in accordance with the Guide for Care and Use of Laboratory Animals published by the NIH. The study protocols were approved by the Animal Care and Use Committee of the *Facultad de Ciencias Bioquímicas y Farmacéuticas de Rosario (UNR)*. Cauda epididymal mouse sperm were collected from C57BL/6 young adult male mice (8–13 weeks old). Each minced cauda epididymis was placed in 500 µl of a modified Krebs-Ringer solution H-TYH HEPES-buffered medium^58^ containing 119.3 mM NaCl, 4.7 mM KCl, 1.2 mM KH_2_PO_4_, 1.2 mM MgSO_4_, 5.6 mM glucose, 0.5 mM sodium pyruvate, 1.7 mM Ca^2+^, and 20 mM HEPES (pH 7.4). The H-TYH medium accounts for non-capacitating medium. After 20 min, epididymides were removed, and the suspension was adjusted with non-capacitating medium to a final concentration of 1–2 × 10^7^ cells/ml. For capacitation, BSA and NaHCO_3_ were added to final concentrations of 5 mg/ml and 15 mM respectively.

### Human Sperm Preparation

Human donors were provided with written information about the study prior to giving informed consent. The study protocol was approved by the Bioethics Committee of the *Instituto de Biología y Medicina Experimental* (IByME, CONICET). The studies are in compliance with the Declaration of Helsinki principles. Semen samples were obtained by masturbation from healthy donors after 3–5 days of abstinence and analyzed following WHO recommendations (World Health Organization. 2010). All samples fulfilled semen parameters (total fluid volume, sperm concentration, motility, viability and morphology) according to WHO normality criteria. Samples were allowed to liquefy for 1 h at room temperature, then, sperm ejaculates were allowed to swim-up in non-capacitating media at 37 °C for 1 hour. The protein extracts were obtained from the motile selected spermatozoa. The non-capacitating medium used was HEPES-buffered human tubal fluid (HTF) containing 90.7 mM NaCl, 4.7 mM KCl, 0.3 mM KH_2_PO_4_, 1.2 mM MgSO_4_, 2.8 mM glucose, 3.4 mM sodium pyruvate, 1.6 mM CaCl_2_, 60 mM sodium lactate and 23.8 mM HEPES (pH 7.4).

### Cell Lines and Media

Protein extracts from CHO cell line were used in this study as positive control for detection of deacetylases, kindly donated by Dolores Campos. Cells were cultured in DMEM medium at 37 °C in a humidified 5% CO_2_ incubator.

### SDS-PAGE and Immunoblotting

After incubation in the different experimental treatments, sperm were collected by centrifugation at 800 × *g* for 4 min and washed in 1 ml of TBS twice. The cell pellet was re-suspended in Laemmli sample buffer without β-mercaptoethanol, vortexed for 5 sec and boiled for 3 min. After centrifugation, 5% β-mercaptoethanol was added to the supernatants and boiled again for 5 minutes. Protein extracts equivalent to 0.5–1 × 10^6^ sperm/lane were subjected to SDS-PAGE and electrotransferred to PVDF membranes (Bio-Rad) at 250 mA for 60 min on ice. Membranes were blocked with 3% BSA in TBS containing 0.1% Tween-20 (T-TBS). Antibodies were diluted in T-TBS as follows: 1/500 for anti-Acetyl Lysines, 1/3,000 for anti-Acetyl αTubulin, 1/3,000 for anti-αTubulin, 1/250 for the different anti-deacetylases, 1/10,000 for anti-pTyr and 1/3,000 for anti-pPKA substrates. Secondary antibodies were diluted 1/15,000 in T-TBS and developed using an enhanced chemiluminescence detection kit (ECL Plus, Amersham Biosciences, GE-Healthcare) according to manufacturer’s instructions. When necessary, PVDF membranes were stripped at 60 °C for 15 min in 2% SDS, 0.74% β-mercaptoethanol, and 62.5 mM Tris (pH 6.5) and washed six times for 5 min each time in T-TBS. In all experiments, molecular masses were expressed in kilodaltons (kDa).

### Immunocytolocalization

Cells were seeded on 8-well glass slides. After air-drying, sperm were fixed with 3.7% paraformaldehyde in PBS for 15 min at room temperature, washed with PBS and permeabilized with 0.5% Triton X-100 for 5 min. After washing with PBS, cells were incubated with 5% BSA in PBS for 1 h at room temperature, and then with the indicated antibodies diluted in 1% BSA in PBS for 3 h. The antibodies were washed with PBS, and the slides were incubated with Hoescht, PSA-FITC, Alexa 546 anti-rabbit and/or Alexa 488 anti-mouse. Before mounting with Slow-Fade Light reagents (Molecular Probes, Eugene, OR), samples were washed with PBS. Images of both conditions (NC and C) were acquired using the Zeiss LSM880 confocal microscope with the same settings and analyzed with ImageJ (v1.38, NIH). Fluorescence intensities corresponding to Acetylated Lysines and Acetylated αTubulin were quantified using ImageJ. Images from both conditions (NC and C) were equally processed. The background was eliminated by adjusting the threshold and the ROIs were set with the wand tool. Each immunocytochemical assay included negative controls, replacing the primary antibody with PBS.

### PKAc immunoprecipitation

Sperm were incubated for 90 min under the indicated conditions. Samples were centrifuged at 1,700 × *g* for 1 min. The resulting pellet was resuspended in RIPA buffer (10 mM Tris-HCl pH 7.2, 50 mM NaCl, 0.1% SDS, 1% Triton X-100, 1 mM EDTA, 20 mM sodium fluoride, 40 mM Glycero-3 phosphate and protease inhibitors), incubated on ice for 30 min and centrifuged at 4 °C for 5 min at 2500 × *g*. Supernatants containing 1 × 10^8^ cells in a final volume of 500 µl were incubated with 3 µg of anti-PKAc antibody for 2 h at RT (23–25 °C) with constant rocking. After the addition of 30 µl of protein G-sepharose (GE Healthcare), the reactions were further rocked for 1 h at RT. The immune complex was recovered by centrifugation, washed four times in RIPA buffer, and subjected to SDS/PAGE and western blot. To avoid immuno-reactive signals from IgG heavy chains, rabbit and mouse anti-IgG light chain were used.

### Membrane Potential Assay in Cell Populations

After treatment, cells were loaded with 1 μM of the membrane-potential-sensitive dye DISC_3_(5) (Molecular Probes) for 2 min. No mitochondrial un-couplers were used because their contribution to the resting potential has been determined to be insignificant^59,60^. Sperm were transferred to a gently stirred cuvette at 37 °C, and the fluorescence was monitored with a Varian Cary Eclipse fluorescence spectrophotometer at 620/670 nm excitation/emission wavelengths. Recordings were initiated when steady-state fluorescence was reached and calibration was performed at the end of each measure by adding 1 μM valinomycin and sequential additions of KCl for internal calibration curves, as previously described^31,59,61^. Sperm membrane potential (*Em*) was obtained from the initial fluorescence (measured as Arbitrary Fluorescence Units) by linearly interpolating it in the theoretical *Em* values from the calibration curve against arbitrary fluorescence units of each trace. This internal calibration for each determination compensates for variables that influence the absolute fluorescence values.

For experiments where CatSper opening was assessed, extracellular Ca^2+^ was chelated using EGTA allowing sodium influx through CatSper to occur. The magnitude of the depolarization caused by Na^+^ influx relates to the extent of channel opening. Cells were loaded with DISC_3_(5) and fluorescence recorded as detailed above. Calcium was chelated with 3.5 mM of EGTA to a value of free calcium of 138 nM (MaxChelator)^62^. The fluorescence change after EGTA addition was presented as (F_EGTA_−F0)/F0 × 100, where F_EGTA_ represents fluorescence intensity after EGTA addition and F0 is the mean of 1 min of acquisition before addition of EGTA^30,31^.

### Live Imaging of Intracellular Ca^2+^ Levels

Non-capacitated sperm were incubated in H-TYH medium containing 2 μM Fluo3-AM (Molecular Probes) and 0.05% Pluronic acid for 20 min at 37 °C before washing by centrifugation at 700 × *g* for 4 min. Once loaded, sperm were immobilized on mouse laminin (0.1 mg/ml)-coated coverslips to allow recordings. The chamber was filled with recording medium (non-capacitating H-TYH) in the absence or presence of 5 μM mibefradil or 30 μM sPKI. Ca^2+^ imaging was performed before, during, and after non-capacitating H-TYH with or without deacetylase inhibitors (iDACs) or capacitating media addition; as a vitality control 20 μM A23187 was added at the end of each experiment. The system used consisted of a Zeiss LSM880 scan head on an axio observer Z1 inverted microscope with a 60 × 1.4 AN oil immersion objective. A laser line 488 nm of an argon ion laser was used for the excitation; the detection was done in a GaAsP spectral detector with a bandwidth between 508 and 588 nm. Bi-directional scanning was used with a Dwell time of 1.03 μsec at 512 × 512 pixels resulting in an acquisition of 3 frames per second for periods of 8–10 minutes. Movies were processed and analyzed in Image J (v1.38, NIH). Fluorescence is expressed as (F-F0)/(Fmax-F0), where (F-F0) was the change in fluorescence signal intensity and F0 the baseline as calculated by averaging 150 frames before stimulus application.

### Sperm Motility Analysis

Sperm suspensions were loaded on a 46-μm deep slide and placed on a microscope stage at 37 °C. Sperm movements were examined using computer-assisted semen analysis (CASA) system (Microptic, SCA evolution). Parameters used were as follows: 30 frames acquired, frame rate of 60 Hz, and cell size of 30–170 μm^2^. At least 20 microscopy fields corresponding to a minimum of 200 sperm were analyzed in each experiment. The following parameters were measured: mean path velocity (VAP, μm/sec), curvilinear velocity (VCL, μm/sec), straight-line velocity (VSL, μm/sec), linearity (LIN, %), amplitude of lateral head displacement (ALH, μm), and straightness (STR, %). Sperm were considered hyperactivated when presenting VCL ≥ 271 μm/sec, LIN < 50%, and ALH ≥ 3.5 μm.

### Acrosomal Status Assays

After incubation in the respective conditions for 60 min, progesterone (20 μM) was then added and incubated for another 30 min. sPKI was added 10 min before the addition of progesterone. Cells were seeded on 8-well glass slides. After air-drying, sperm were fixed with 3.7% formaldehyde in PBS for 15 min at room temperature, permeabilized with 0.5% Triton X-100 for 5 min, washed with PBS and incubated with PBS containing 1% BSA and FITC-conjugated pisum sativum lectin (1/500) for 1 h at room temperature. Before mounting samples were washed with PBS (four times for 5 min each time). Epifluorescence microscopy was used to assess acrosomal status. At least 200 sperm were analyzed in each condition.

### Statistical analysis

Data are expressed as mean ± standard error of the mean (SEM) of at least three independent experiments for all determinations. Statistical analyses were performed using the GraphPad Prism 6 software (La Jolla, CA USA). Student’s *t* test was used to compare mean values between control and tested groups, while the difference between mean values of multiple groups was analyzed by one-way analysis of variance (ANOVA) with multiple comparison tests. A probability (P) value P < 0.05 was considered statistically significant.

## Electronic supplementary material

Movie S1

Movie S2

Movie S3

Movie S4

Movie S5

Supplementary Information

## Data Availability

Data generated during the current study are available from the corresponding author upon request.
