# Supplementary material for: Lysine acetylation modulates mouse sperm capacitation
Source: Sci Rep. 2018 Sep 6;8:13334. doi: 10.1038/s41598-018-31557-5 (PMC6127136; doi:10.1038/s41598-018-31557-5)
Supplement: Supplementary file 6 — Supplementary Information [file 41598_2018_31557_MOESM6_ESM.pdf]

Lysine acetylation modulates mouse sperm capacitation

Carla Ritagliati[1], Guillermina M. Luque[2], Cintia Stival[1], Carolina Baro Graf[1], Mariano G. Buffone[2] and Dario Krapf[1*].

[1] Laboratory of Cell Signal Transduction Networks, Instituto de Biología Molecular y Celular de Rosario (IBR), CONICET-UNR, Rosario 2000, Argentina.

[2] Laboratory of Cellular and Molecular Reproductive Biology, Instituto de Biología y Medicina Experimental (IBYME), CONICET, Buenos Aires C1428ADN, Argentina.

*To whom correspondence should be addressed: Dario Krapf, Instituto de Biología Molecular y Celular de Rosario, CONICET-UNR; Ocampo y Esmeralda. Rosario 2000, SF, Argentina; +54 (341) 423-7070 ext 654. mail: krapf@ibr-conicet.gov.ar

SUPPLEMENTARY FIGURES

Figure S1

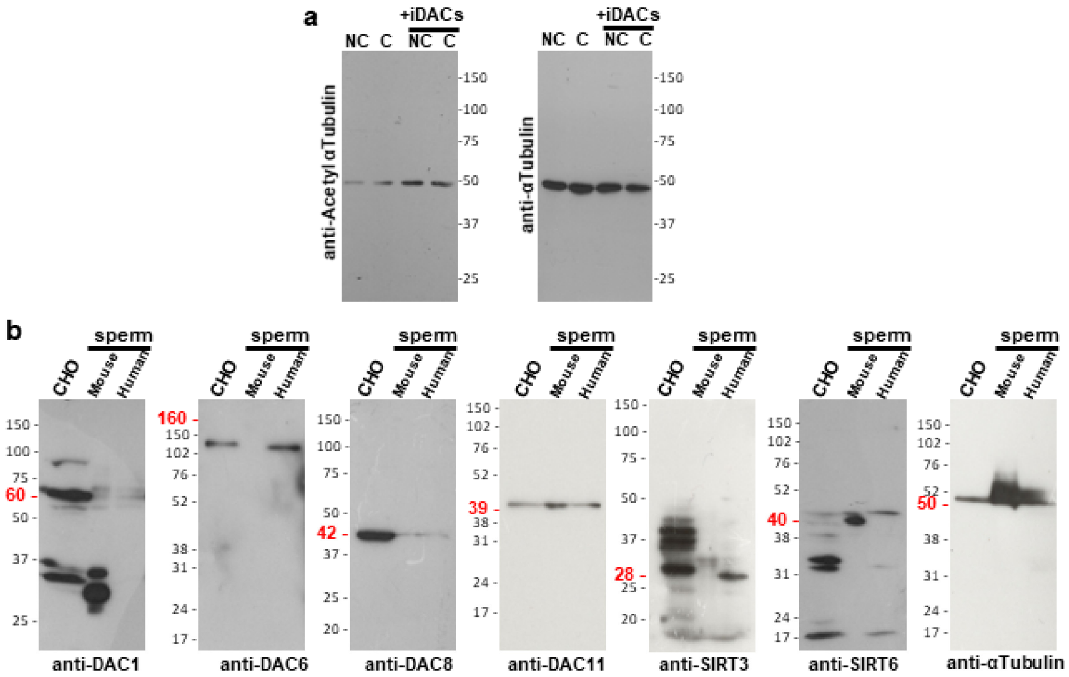

Figure S1: Full length blots from cropped blots in Figure 1. **a,** Western blot analysis of non-capacitated (NC) and capacitated (C) sperm in the absence or presence of +iDACs (5 µM TSA and 0.5 mM NAM) using anti-Acetyl αTubulin and anti-αTubulin antibodies. **b,** Western blot analysis of total protein extracts from *in vitro* CHO cell cultures, mouse sperm and human sperm, with antibodies against the following deacetylases: DAC1, DAC6, DAC8, DAC11, SIRT3 and SIRT6. anti-αTubulin was used as a loading control. Molecular weights are indicated in kDa. The expected molecular weights are indicated in red.

Figure S2

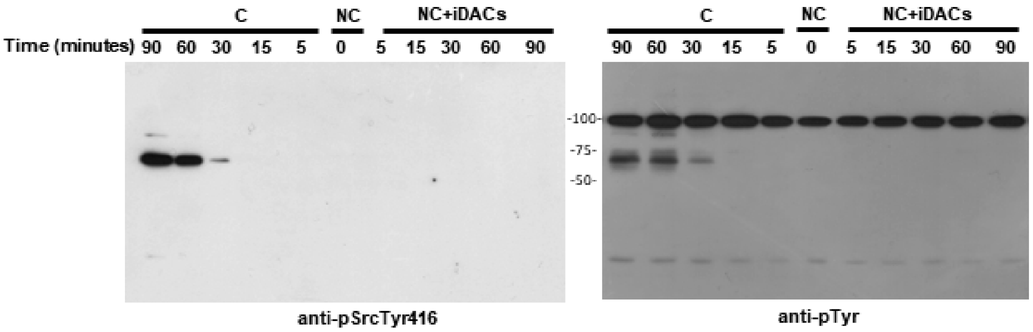

Figure S2: Full length blots from cropped blots in Figure 5e. Western blot analysis using anti-pSrcTyr416 and anti-pTyr of sperm incubated in capacitating (C) or non-capacitating conditions with iDACs (NC+iDACs) (5 µM TSA and 0.5 mM NAM) for different times. Molecular weights are indicated in kDa. Tyrosine phosphorylated Hexokinase (95 kDa) served as a loading control.

SUPPLEMENTARY MOVIES LEGENDS

Movie S1. Single-cell Ca^{2+} imaging. Intracellular Ca^{2+} concentration imaging of non-capacitated sperm loaded with Fluo3-AM on laminin-coated coverslips. Fluorescence recordings were obtained at 3 Hz as indicated in Methods. Non-capacitating medium with vehicle (+vehicle) was added a minute after recording started and A23187 (+IONO, 20 µM) at the end.

Movie S2. Single-cell Ca^{2+} imaging. Intracellular Ca^{2+} concentration imaging of non-capacitated sperm loaded with Fluo3-AM on laminin-coated coverslips. Fluorescence recordings were obtained at 3 Hz as indicated in Methods. Non-capacitating medium with deacetylase inhibitors (+iDACs) was added a minute after recording started and A23187 (+IONO, 20 µM) at the end.

Movie S3. Single-cell Ca^{2+} imaging. Intracellular Ca^{2+} concentration imaging of non-

capacitated sperm loaded with Fluo-3AM on laminin-coated coverslips. Before the recording, the cells were incubated for 10 min with 5 μM Mibefradil (+mib). Fluorescence recordings were obtained at 3 Hz as indicated in Methods. Non-capacitating medium with deacetylase inhibitors (+iDACs) was added a minute after recording started and A23187 (+IONO, 20 μM) at the end.

Movie S4. Single-cell Ca^{2+} imaging. Intracellular Ca^{2+} concentration imaging of non-capacitated sperm loaded with Fluo-3AM on laminin-coated coverslips. Before the recording, the cells were incubated for 10 min with 30 μM sPKI (+sPKI). Fluorescence recordings were obtained at 3 Hz as indicated in Methods. Non-capacitating medium with deacetylase inhibitors (+iDACs) was added a minute after recording started and A23187 (+IONO, 20 μM) at the end.

Movie S5. Single-cell Ca^{2+} imaging. Intracellular Ca^{2+} concentration imaging of non-capacitated sperm loaded with Fluo-3AM on laminin-coated coverslips. Fluorescence recordings were obtained at 3 Hz as indicated in Methods. Capacitating medium (+HCO_3^-/BSA) was added a minute after recording started and A23187 (+IONO, 20 μM) at the end.
